# Impulsivity as a predictor factor of health-related risk-taking

**DOI:** 10.1192/j.eurpsy.2024.1353

**Published:** 2024-08-27

**Authors:** A. Megías-Robles, L. Moreno-Ríos, R. Megías-Robles, A. Martínez-Estrella, M. A. Torres

**Affiliations:** ^1^Department of Basic Psychology, University of Malaga, Málaga; ^2^Junta de Andalucía, Motril; ^3^Department of Experimental Psychology, University of Granada, Granada, Spain

## Abstract

**Introduction:**

There is a broad consensus that risk taking is largely determined by risk perception. However, previous literature has shown numerous examples of situations associated with potential health risks where our decisions are not made in accordance with the level of perceived risk.

**Objectives:**

The aim of the present research was to investigate the role of impulsivity in the explanation of the discordance observed between risk perception and risk-taking in health-related domains.

**Methods:**

The sample consisted of 612 participants (Mage = 23.54, 73,2% women). All participants were assessed for levels impulsivity and levels of risk perception and risk-taking propensity in contexts related to health.

**Results:**

Results revealed that higher levels of impulsivity were significantly related to a lower tendency to perceive and take risks in the health domain. Most important for our objectives, we observed that the relationship with impulsivity was significantly stronger for risk taking than for risk perception. Moreover, impulsivity significantly predicted risk taking propensity when controlling for risk perception.

**Conclusions:**

These findings suggest that, in the health-related domains, impulsivity can differentially affect risk perception and risk taking, thus, offering a possible explanation for the inconsistencies observed in the previous literature.

**Disclosure of Interest:**

None Declared

